# Male Genital Mutilation in the High-Mountain Goblin Spider, *Unicorn catleyi*

**DOI:** 10.1673/031.011.11801

**Published:** 2011-09-14

**Authors:** Matías A. Izquierdo, Gonzalo D. Rubio

**Affiliations:** ^1^ CONICET. División de Aracnología, Museo Argentino de Ciencias Naturales, Av. Ángel Gallardo 470, C1405DJR Buenos Aires, Argentina; ^2^ CONICET. Diversidad Animal I, Facultad de Ciencias Exactas, Físicas y Naturales, Universidad Nacional de Córdoba, Av. Vélez Sarsfield 299, X5000JJC Córdoba, Argentina

**Keywords:** female genitalia, Haplogynae, mating plugs, Oonopidae, spider reproduction

## Abstract

Male genital mutilation is a common mechanism by which males reduce sperm competition by plugging female insemination ducts with different parts of its own genital system. This behavior is frequent in many spider families but is uncommon in Haplogynae. The reproductive biology of Dysderoidea is not well studied and the data is fragmentary; male genital mutilation has been reported only for one species of Oonopidae. This study provides evidence of male genital mutilation in *Unicorn catleyi* Platnick and Brescovit (Araneae: Oonopidae). Pieces of the embolus were found in the female posterior receptaculum. This behavior is a strategy used by the males in order to guarantee their paternity and not for escape from female attacks as has been reported for other species of Araneae, since cannibalism is unlikely in this species. The presence of embolus in the posterior receptaculum suggests this is the first place where sperm is received. The similarity of the female genitalia of *U. catleyi* to those of Orsolobidae, along with sclerotization of the seminal duct in the male copulatory bulb that is also present in *Orchestina, Xiombarg*, and Orsolobidae, provide strong evidence of the basal position of this genus in the family Oonopidae.

## Introduction

Spider reproduction comprises a wide variety of morphological and behavioral strategies that include male sacrifice, production of mating plugs, extreme sexual size dimorphism, and polyandrous females (Nessler et al. 2007a; Miller 2007; Uhl et al. 2010). After mating, males of some animals, including arthropods and nematodes, deposit a mating plug that is thought to prevent or reduce intromission by other males. In this way, males increase the likelihood of their paternity (Jackson 1980; Robinson 1982; Matsumoto and Suzuki 1992; Barker 1994; Simmons 2001; Aisenberg and Eberhard 2009; Peretti 2010). In spiders, plugs might be formed by secretions generated by accessory glands in the male palp or female genital tract (Exline and Levi 1962; Leopold 1976; Masumoto 1993; Elgar 1998; Uhl et al. 2010), while in other cases are formed by fragments of male copulatory organs or even entire male palps that break off during copulation and remain in the insemination duct of the female (Levi 1968, 1983; Nessler et al. 2007b). Plugs have been reported in at least 41 families, some of which are not phylogenetically related groups (Uhl et al. 2010).

Embolus tips act as mating plugs in some species. It has been proposed that the breakage of the male pedipalp may facilitate male survival from the regular female cannibalistic attacks, since ectomizing a part of the palp may allow the male to quickly jump off the female immediately after copulation (Nessler et al. 2007b). Alternatively, mating plugs are considered an adaptive strategy to reduce sperm competition in order to guarantee paternity (Austad 1982; Nessler et al. 2007b; Uhl et al. 2010). Mating plugs are not frequent in haplogine spiders and only one case has been reported for Dysderoidea in the family Oonopidae (Platnick and Dupérré 2009).

The spiders of the genus *Unicorn* Platnick & Brescovit (Araneae: Oonopidae) are relatively large oonopids from South America, with a total length of 2.2–2.8 mm. The genus includes six species known from Chile, Bolivia, and semi-desert areas of western Argentina. The genus presents sexual dimorphism in some characteristics; males have a clypeal horn and an expanded palpal tibia but no sexual differences are observed in the body size. Virtually nothing is known about the natural history of *Unicorn*. They are uncommon in collections and are difficult to find. With the exception of *U. socos* collected at 469 meters, the genus is distributed at high elevations from 1100–3780 meters, where the dominant physiognomy is the semi-desert biome. It has been proposed that this genus, along with the genera *Orchestina* and *Xiombarg*, is probably one of the most basal members of the Oonopidae (Platnick and Brescovit 1995).

The purpose of this paper is to report and describe the male genital mutilation in the genus *Unicorn* from observations of specimens of *U. catleyi* Platnick and Brescovit (Araneae: Oonopidae) discussing its probable function in the species. Also, brief additional descriptions of the male and female genitalia are provided using scanning electron microscopy.

## Materials and Methods

### Material examined

Collections of *Unicorn catleyi*. Salta province, Argentina, road to Muñiano, route 51 between Santa Rosa de Tastil and Muñiano, elevation 3100–4000 MASL, 22 August 2006, G.D. Rubio collector, pitfall traps 2 males, 2 females, preserved in the Museo Argentino de Ciencias Naturales “Bernardino Rivadavia”, Buenos Aires (CL Scioscia) (MACN-Ar 22099, PBI_OON 00015060, preparation codes MAI 270, 271, 279–281, 300, 328). Same data 15 November 2006, 1 male (MACN-Ar 22100, PBI_OON 00015059). In order to explore the male palp morphology in species of the same genus, additional material was examined: *Unicorn argentina*. Mendoza province, Argentina: Ñacuñan reserve, 20 October 1997. S Lagos collector, pitfall traps; 2 males, 1 female, (PBI_OON 00015084, preparation codes MAI 327) in Instituto Argentino de Investigaciones de las Zonas Áridas (IADIZA, Entomology, Sergio A Roig).

The description of the male and female genitalia generally follows that of Platnick and Brescovit (1995) and Forster and Platnick (1985). Male and female genital systems were dissected, embedded in clove oil, mounted on a slide, and observed under an Olympus BH-2 compound microscope (Olympus, www.olympus.com). Photographs of compound microscope and stereomicroscope preparations were made with Nikon DXM1200 (Nikon, www.nikon.com) and Leica DFC 290 (www.leicamicrosystems.com) digital cameras, and the focal planes combined with Helicon Focus 3.10.3 and 4.62 Pro (www.heliconsoft.com). The genitalia of one female was digested in a borax-pancreatin solution for 1–2 hours according to the protocol of Alvarez-Padilla and Hormiga (2007), and then flushed with a micropipette in distilled water until all the soft tissues were removed. Genitalia were then observed in a lactic acid medium using a compound microscope. For SEM observations, two male palps and the digested female genitalia were dehydrated in a graded ethanol series (80–100%), critical point dried, and Au-Pd coated. SEM micrographs were taken under high vacuum with a FEI XL30 TMP (FEI, www.fei.com).

**Figure 1.**
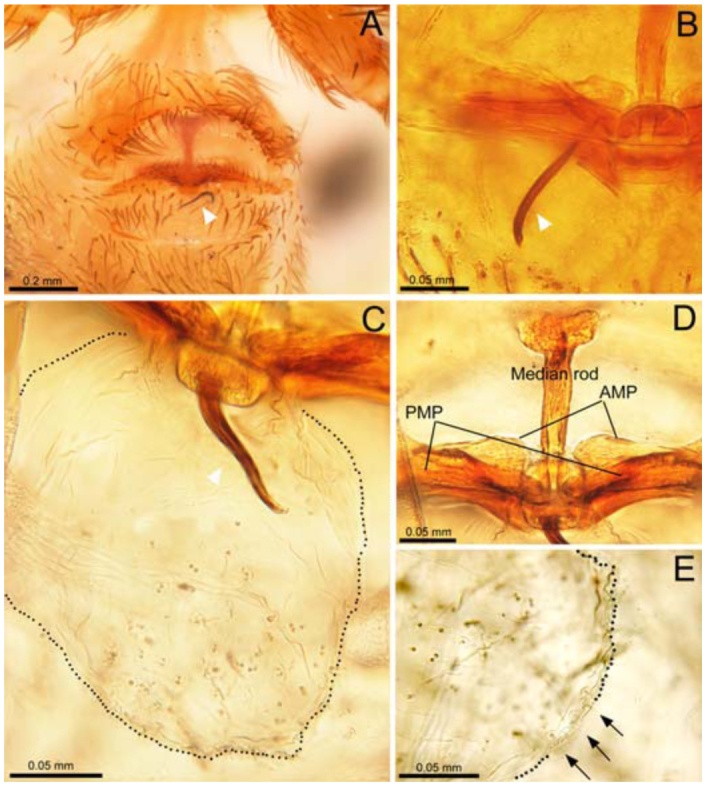
*Unicorn catleyi*, female genitalia. (A) Epigastric region in ventral view; (B) internal genitalia cleared in a clove oil in dorsal view; (C) digested genitalia in lactic acid; (D) anterior elements of the genitalia; (E) posterior receptaculum. Arrowheads point to the embolus tip, black arrows point to the gland ducts of the posterior receptaculum, black dots delineate the margins of the posterior receptaculum. AMP, anterior median plate; PMP, posterior median plate. High quality figures are available online.

## Results

The females examined contained an embolus tip inside the posterior receptaculum; in one specimen the embolus was observed across the abdominal cuticle through the transparent (Figure 1A). Only after the complete digestion of the genitalia was it possible to observe the embolus tip inside the posterior receptaculum. Because of the cleaning process during dissection, the original position of the embolus (Figure 1B) changed slightly inside the posterior receptaculum (Figure 1C).

**Figure 2.**
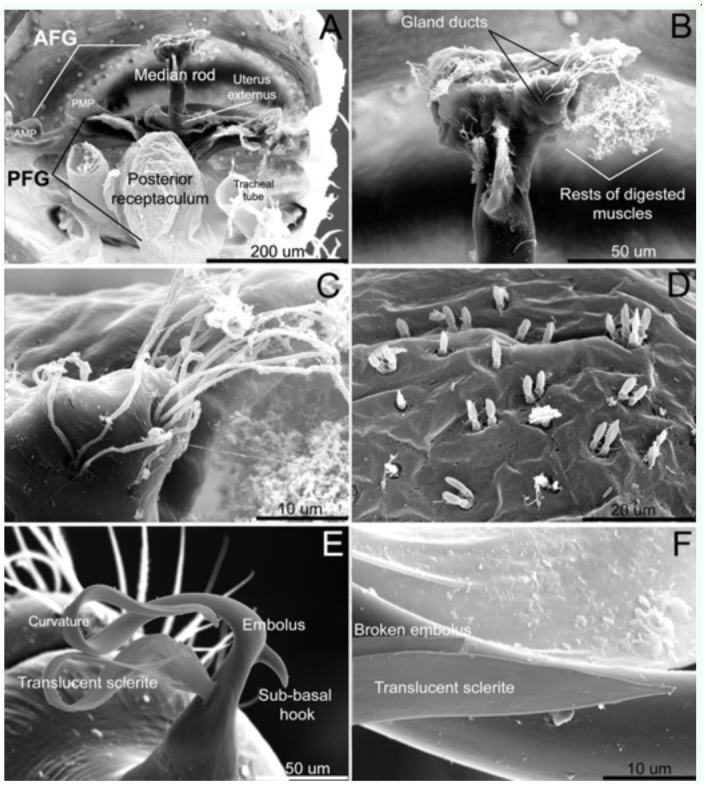
*Unicorn catleyi*, male and female genitalia in SEM images. AD: female genitalia, (A) dorsal view; (B) apical portion of the anterior median rod; (C) gland ducts of the anterior median rod; (D) gland ducts of the posterior receptaculum. E–F: male genitalia, (E) entire embolus and translucent sclerite; (F) broken embolus and translucent sclerite. AFG, anterior portion of the female genitalia; AMP, anterior median plate; PFG, posterior portion of the female genitalia; PMP, posterior median plate. High quality figures are available online.

### Female genitalia

The anterior portion of the female genitalia (Figure 2A) is a highly sclerotized structure formed by a conspicuous median rod and an anterior median plate extended to both right and left sides (Figures 1D, 2A). The median rod bears many gland ducts and the remainder of digested muscles near the tip (Figures 2B, 2C). The posterior portion of the female genitalia is shown in Figure 2A with a posterior median plate extended to both left and right sides (Figures 1D, 2A) and a globose, membranous receptaculum bearing many gland ducts on its surface (Figures 1A, 1E). The uterus externus is located between the base of a median rod and the posterior median plate (Figure 2A).

**Figure 3.**
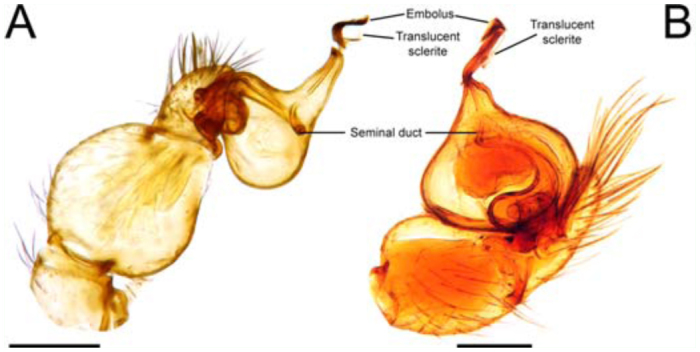
Male copulatory bulb of *Unicorn* spp. cleared in a clove oil. (A) *Unicorn catleyi*; (B) *U. argentina.* Scale bars = 0.2 µm. High quality figures are available online.

### Male palp

The embolus with sub-basal hook, long and describing a pronounced curve at the tip is shown in Figure 2E. The SEM images reveal that the male embolus lacks a suture line were it could break off during copulation. The curvature in the embolus might act as a lever mechanism allowing the embolus to break off at this point. However the breakage also occurs ahead of this curvature, near the tip of the embolus (Figure 2F). There is a translucent sclerite originating near the embolus base (Figure 2E). Because of the translucent nature of this sclerite, it can be more or less joined to the embolus and can sometimes be placed in different positions making it difficult to detect. The male copulatory bulb has a sclerotized seminal duct (Figure 3A). This character was also confirmed by the examination of specimens of *Unicorn argentina* (Mello-Leitão 1940) (Figure 3B). The male palp drawings of Platnick and Brescovit (1995) suggest the presence of a seminal duct in the other species of the genus. Also, the seminal duct is present in the genus *Orchestina* (Saaristo and van Harten 2006, Izquierdo personal observation) and *Xiombarg* as revealed in the original drawing of Brignoli (1979).

## Discussion

### Male genital mutilation and its possible functions

Two studies regarding the occurrence of plugs support the hypothesis that the plug provides paternity protection. Miller (2007) suggests that mating plugs formed from fragments of male genitalia are an adaptive mechanism when post-mating male-male competition is intensive. Studies on *A. bruennichi* are inconsistent with the alternative hypothesis that the damage occurs as the male attempts to escape attack by the female (Nessler et al. 2007b).

More observations of behavior are needed to make accurate predictions of the sexual strategies of this species. This issue represents a challenge since the species is difficult to find in the field, and indirect methods, such as pitfall traps, are often needed to collect them. Given this, we can only make assumptions or predictions in analyzing the genital morphology of the specimens. Our observations could be consistent with the ideas of Miller (2007) and Nessler et al. (2007b). Cannibalistic attacks are more evident in those arthropod species with remarkable body size dimorphism (Wilder and Rypstra 2008). In general, female spiders are larger than the cannibalized males. Although cannibalism in *U. catleyi* should not be dismissed, this behavior might be rare since males and females are not sexually dimorphic in body size. Additional data in favor of this hypothesis is that males with the broken embolus in only one palp were found in pitfall traps, suggesting that they remain alive after copulation. In addition, the presence of only one embolus fragment in the female genital tract of this species may reflect the efficiency of the embolus tip in preventing future insertions by the palp of other males. However, more specimens are needed to make predictions of greater accuracy.

Predictions of the pattern of sperm priority are difficult to make; many more specimens need to be studied. In most haplogyne spiders, the female genitalia exhibit a dead end design (Uhl 2002). These spiders should exhibit last male sperm priority, as the last sperm to enter should lie closest to the single fertilization duct. If there is no mixing of sperm from several males, then this arrangement of the female genitalia is a disadvantage for the first mating males. If this hypothesis is correct, the formation of a mating plug by the first mating males could represent an adaptive switch by which these males have an advantage over the second males. If mating with the first males only is indeed the norm in *U. catleyi*, as suggested by the discovery of single embolus tips in females, then first males not only overcome the morphological difficulties of the female genitalia but also may increase paternity. The exactly place where the sperm transference takes place has been difficult to determine in several species of Dysderoidea. However, the presence of the male embolus inside the posterior receptaculum might suggest that this is the place where the sperm transference occurs.

Some details of reproductive strategies have been documented in related species of Dysderoidea. Platnick and Dupérré (2009) reported the presence of an embolus fragment in the female genitalia of one species of the oonopid genus *Scaphiella,* while Řezác (2009) described the traumatic insemination in *Harpactea sadistica* in which males inseminate females with its needle-like intromittent organs by penetrating the female abdomen wall. In addition, Burger (2007) reported sperm dumping in the oonopid *Silhouettella loricatula* (Roewer 1942). As we see, the complexities in reproductive strategies and in the female genital morphology seem to be more abundant than expected for this haplogyne group of spiders.

### Phylogenethic relationships

The female genitalia of *U. catleyi* resembles those described for orsolobids, such as *Orsolobus pucara* (see Izquierdo and Labarque 2010). In both species there is an anterior median, highly sclerotized sclerite, or median rod, with gland ducts on its surface and lateral plates. This sclerite serves as an attachment for muscles that might have a function in some mechanisms of sexual behavior such as sperm dumping, as has been described for the oonopid *Silhouettella loricatula* (Burger et al. 2006). Also, the gland ducts of the posterior receptaculum are extremely similar to those found in *O. pucara*. Because of the presence of gland ducts on the anterior median rod, Izquierdo and Labarque (2010) hypothesize the possible homology between this structure and the membranous anterior receptaculum found in Segestriidae and Dysderidae. It is possible that the anterior receptaculum has evolved to a sclerotized structure in order to provide attachment sites for muscles. In Oonopidae the sclerotized anterior receptaculum with a median rod is present in at least *Orchestina, Xiombarg*, and non-described species of soft-bodied oonopids (Cristian Grismado, personal communication), while it is a highly derived structure in other genera such as *Opopaea* (see Burger et al. 2003).

The sclerotization of the seminal duct in the male copulatory bulb in *Unicorn* and *Orchestina*, added to the similarity of the female genitalia of these genera with those of orsolobids, further supports its basal phylogenetic position in the Oonopidae, as has been proposed by Platnick and Brescovit (1995).

## Abbreviations:

**AFG**,: anterior portion of the female genitalia;
**AMP**,: anterior median plate;
**PFG**,: posterior portion of the female genitalia;
**PMP**,: posterior median plate
